# Effects of COVID-19 pandemic on ART Service delivery: perspectives of healthcare workers in a Teaching Hospital in Ghana

**DOI:** 10.1186/s12913-021-07330-2

**Published:** 2021-12-02

**Authors:** Susanna Aba Abraham, Gifty Osei Berchie, Patience Fakornam Doe, Elizabeth Agyare, Stephen Ayisi Addo, Dorcas Obiri-Yeboah

**Affiliations:** 1 Department of Adult Health, School of Nursing and Midwifery, College of Health and Allied Sciences, University of Cape Coast, Cape Coast, Ghana; 2 Maternal and Child Health Department, School of Nursing and Midwifery, University of Cape Coast, Cape Coast, Ghana; 3 Clinical Microbiology/Public Health Unit, Cape Coast Teaching Hospital, Cape Coast, Ghana; 4 Programme Manager, National HIV/AIDS Control Programme, Korle-Bu, Accra, Ghana; 5 Microbiology and Immunology Department, School of Medical Sciences, College of Health and Allied Sciences, University of Cape Coast, Cape Coast, Ghana; 6 Directorate of Research Innovation and Consultancy, University of Cape Coast, Cape Coast, Ghana

**Keywords:** COVID-19, Pandemic, ART, Healthcare workers, Ghana, HIV

## Abstract

**Background:**

Ghana has a generalized HIV epidemic and efforts have been made to curb the spread and reverse its effects on the general population. In the wake of COVID-19 pandemic, the health system was unsettled and antiretroviral therapy (ART) care has been impacted in diverse ways. The study sought to explore the effects of COVID-19 on ART service provision in Ghana from the perspectives of the healthcare workers.

**Methods:**

An exploratory-descriptive qualitative approach was employed in this study. Using maximum variation sampling method, fifteen healthcare workers; nurses, data managers and pharmacists were recruited from an ART clinic in a Teaching Hospital in Ghana. In-depth interviews were conducted and analysed using Braun and Clarke thematic approach.

**Results:**

Three themes emerged from the data; “… And the pandemic struck”, “Impact of the pandemic on ART service delivery”; “Effecting the needed change”. The healthcare workers’ initial reactions to the pandemic and their show of commitment in ensuring continued ART service was evident. COVID-19 impacted service delivery in three main ways; (1) clients’ clinic attendance was erratic at various stages of the pandemic, (2) irregular resource availability as shortage was reported due to affected last mile delivery as a result of the lockdown in Accra, and (3) the health worker-patient interaction became less engaging because of established COVID-19 protocols. The healthcare workers however instituted strategies such as adjusting the patient appointment schedule, health professionals’ work schedule, establishing several work stations, task-shifting, and ensuring the implementation of all the COVID-19 protocols within the ART unit to ensure consistent service delivery as well as patient and staff safety. The study also found a decline in the implementation of several strategies established in the ART clinic during the initial phases of the pandemic such as a decline in the supply of *Personal Protective Equipment (PPEs)* by hospital management.

**Conclusions:**

Although several strategies were implemented to manage the effects of the pandemic on ART care, there is a need to establish pathways of support for healthcare workers within the ART clinic and to consolidate as well as institutionalise the changes that ensured continuous but safe service delivery.

## Background

The Joint United Nations Programme on HIV/AIDS (UNAIDS) estimated 1.7 million people were newly infected with HIV in 2019 alone [1]. Ghana, one of the 22 countries carrying the substantial burden of HIV [2] committed to the UNAIDS call to eliminate AIDS by 2030 with the focus on reducing new HIV infections by 80% and AIDS-related death by 80% [3]. Identification of HIV positive persons and enrolling them on Antiretroviral Therapy (ART) is essential to achieving the target [3].

While strategizing to strengthen the health system towards achieving this goal [3], the world woke up to coronavirus outbreak [4]. By March, 2020, the World Health Organization (WHO) had declared COVID-19 as an pandemic [5]. Ghana was not exempted from the ravaging effects of the pandemic and ranks as the third most impacted country in the WHO Africa region [6]. In curbing its effects, Ghana like several countries instituted a ban on social gathering, tracing infected persons and their contacts, and lockdown in Accra and Kumasi; the biggest cities in Ghana, from where supplies are commonly picked [6].

The rapid infectivity of the coronavirus however unsettled the health systems with a high proportion of infected patients requiring hospitalization [7]. The pandemic also affected HIV/ART services as several studies reported negative psychological effects on healthcare workers [8, 9], decline in clinic attendance [10], and challenges organising services within facilities [11] which further impacted uptake and delivery of ART services.

While attention was primarily focused on reducing the spread of COVID-19, the UNAIDS observes that the global efforts towards combating HIV/AIDS has been disrupted by the COVID-19 pandemic [12]. For ART services, any disruptions would impact the attainment of the 90–90-90 targets. Hence, this study was conducted to unearth the impact of the pandemic on ART service delivery in Ghana, identify lessons learnt to improve service provision as well as ensure adequate preparation for any future pandemics.

## Methods

### The study adopted an exploratory-descriptive qualitative approach

The data collection for the study was conducted between January and March, 2021. The population comprised of nurses, data managers and pharmacists who provided care in the ART clinic at the Cape Coast Teaching Hospital in Ghana. The hospital was the first to initiate antiretroviral therapy in the Central Region of Ghana. It has adequate facilities, staff trained by the National AIDS Control Programme (NACP), and about 3000 patients and serves as the referral centre for all HIV clinics in the region and beyond.

The participants were sampled using maximum variation method to ensure that the sample was drawn from the various categories of healthcare workers, and with different years of experience in ART service provision. Fifteen healthcare workers who had provided care in the ART clinic before at least 1 year before the pandemic and during the COVID-19 pandemic were interviewed individually in an office within the clinic using in-depth techniques. Covid-19 protocols such as social distancing and wearing of nose mask were maintained throughout the interviews.

The interviews lasted between 35 to 45 min and were conducted in English as all the participants were fluent. Data saturation was attained at the twelfth interview as no new information was attained. Three additional interviews were then conducted to confirm that there were enough interviews to information the study [13]*.*

The interviews were audio-recorded and data analysis was guided by Braun and Clarke thematic analysis approach [14] which began with familiarization with the data by reading the printed transcripts and listening to the audio-versions concurrently. The transcripts were then coded by highlighting phrases that were relevant to the topic under study. The highlighted data were then collated together into groups identified by the codes to get a condensed overview of the main points recurring throughout the narratives. Thereafter, themes were generated by identifying patterns among the codes. The themes were then reviewed to ensure an accurate representation of the participants narratives. Finally, the emergent themes were defined by formulating their meaning in relation to the data set.

To ensure rigor, an audit trail was maintained throughout data collection and analysis while member checking was conducted prior to finalisation of the report. Ethical approval was obtained from the Cape Coast Teaching Hospital Ethics Review Committee (CCTHERB/EC/2020/107). The study also complied with all the ethical considerations stipulated in the Declaration of Helsinki.

## Results

### Sociodemographic characteristics

Majority of the participants were females (*n* = 10/15), 40 years or younger (*n* = 13/15) and all of them (*n* = 15/15) had a tertiary education with the least qualification being a diploma. Most of the participants were community health nurses (*n* = 12/15) and the person with the highest experience of providing care in the ART clinic to persons living with HIV had worked for 15 years. The participants had an aggregated 77 years and 5 months of experience working in the ART clinic. Table 1 presents the sociodemographic profile of the participants.

**Table 1:** Socio-demographic characteristics of participants

Interview no.	Pseudonym	Age (in years)	Sex	Qualification	Profession	Experience in ART (Years)
1	Esi	31	F	Diploma	Nurse	9
2	Jessica	29	F	Diploma	Nurse	2
3	Efua	38	F	Bachelors	Nurse	13
4	Angel	32	F	Diploma	Nurse	10
5	Kwesi	27	M	Diploma	Nurse	2
6	Araba	33	F	Bachelors	Data Manager	5
7	Da Vinci	28	M	Diploma	Nurse	3
8	Maame	27	F	Diploma	Nurse	2
9	Aaron	45	M	BSc	Pharmacist	3
10	Kwesi	43	M	HND	Dispensing Technician	15
11	Melissa	26	F	Diploma	Nurse	2
12	Alberta	34	F	Diploma	Nurse	6
13	Mary	39	F	Advanced Diploma	Nurse	3
14	Attah	32	M	Diploma	Nurse	1
15	Sandy	27	F	Diploma	Nurse	1.5 years

### Emergent themes

**Fig. 1:**
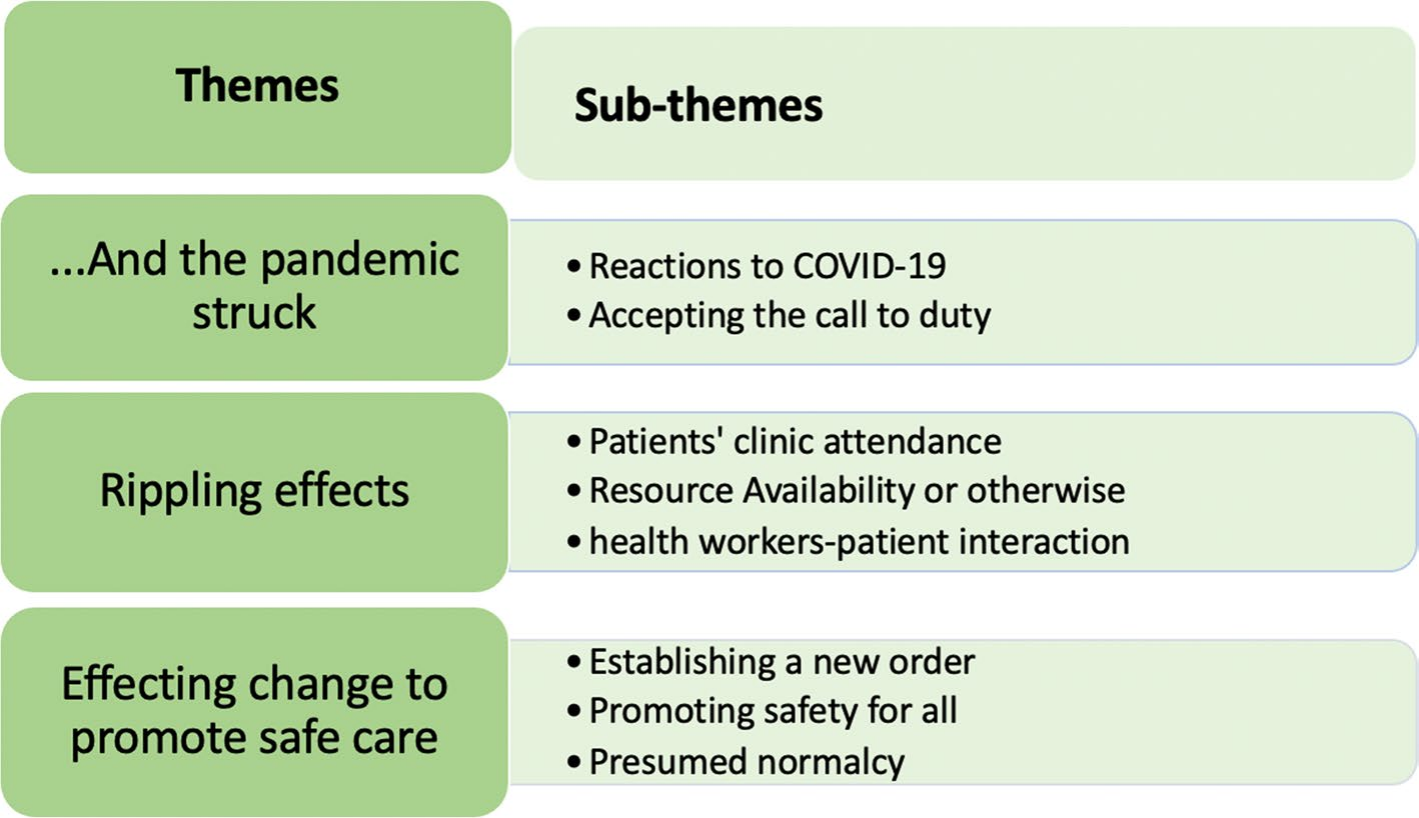
Emergent themes and Sub-themes

Three main themes emerged from the data: “… And the pandemic struck”, “Impact of the pandemic on HIV service delivery” and “Effecting the needed change”. Subthemes were generated under each major theme. The themes and sub-themes are displayed at in Fig. 1 below.

### Theme 1: “… And the pandemic struck”

The sudden emergence of COVID-19 and its declaration as a pandemic impacted health care delivery. The theme describes the healthcare workers’ reactions to the pandemic which reflected unpreparedness but also revealed their acceptance to the call of duty.

### Reactions to COVID-19 pandemic

The narratives revealed varied reactions to the announcement of the pandemic. The initial reaction of the healthcare workers upon the announcement of a case recorded in their health facility was fear. Esi said: *“*In the beginning, it was quite scary working in the [ART] unit.” (Nurse Esi, 9 years in ART care).

Most of the participants said they struggled psychologically because of their knowledge of COVID-19 and its rate of infectivity. “As for psychologically, it wasn’t that easy to handle. Because, you know very well as a health worker the implications of whatever is going on.” (Nurse Jessica, 2 years in ART care).

For others however, the fear was underscored by the inconsistency of information published in the media. Angel explained that:

I was afraid because initially we didn’t have much information on the disease. Even, they [the scientists] were not consistent about the mode of transmission; sometimes they said airborne, other times it was through droplets. Several myths about the disease were aired on the radio. So, we were a little bit confused and afraid (Nurse Angel, 10 years in ART care).

For some participants, concern for their families’ safety heightened their fear of working in the unit during the pandemic. “My fear was should I get exposed, those I live with at home … I was just scared that in case I pick something [the virus] from the [ART] unit, eventually my kid sister will be affected and other people as well.” (Nurse Jessica, 2 years in ART care).

These factors underscored the participants reaction to working in the HIV unit during the pandemic. A participant said “Initially, we were all afraid that we could get it ...We were afraid of coming to work or even going to the ward.” (Nurse Esi, 13 years in ART care).

### Accepting the call to duty

In spite of the healthcare workers initial reactions to the pandemic, the narratives revealed that they showed commitment to providing care to their clients at the ART unit. Some participants indicated relying on their innate strength to continue service delivery. Kwesi said “I gathered courage to do what I was supposed to do for them [HIV clients], like normal service delivery. I just had to do it.” (Nurse Kwesi, 2 years in ART care).

It emerged from the data that some participants committed financially and took steps physiologically to protect themselves while drawing strength spiritually to ensure their safety. Some participants explained that:

We were buying the face mask ourselves and we tried our best that to render the best of service to our clients. We accepted that it was not easy and we just had to pray that COVID-19 will be eradicated so that we could live a normal life. (Nurse Jessica, 2 years in ART care)

We were also advised to provide our own PPE's [Personal protective equipment] along the line. The President even advised us to take care of ourselves … So once in a while I have been taking Vitamin C and Zinc tablets. (Dispensing Technician Akwasi, 15 years in ART care)

The narratives revealed that the healthcare workers opted to report early to work to ensure patients received care as early as possible. Da Vinci said “We decided that instead of starting work at 8am … Some staff come here as early as 6:30am to start with the digital activation of cards.” (Nurse Da Vinci, 3 years in ART care). Nurse angel also reiterated that “During that time [COVID-19 pandemic], the doctors too come earlier than the time they were reporting prior to the pandemic.” (Nurse Angel, 10 years in ART care).

Participants also indicated that the sense of camaraderie among the healthcare workers was enhanced:

We [male nurses] also decided to take over some of the workload for our female colleagues. So, we did a schedule for the breastfeeding nurses as well as those with chronic conditions. So that they had fewer days then was expected in the hospital.

(Nurse Da Vinci, 3 years in ART care)

### Theme 2: the rippling effect of the pandemic

From the narratives, it was evident that fear of COVID-19 impacted service delivery in three ways; “Patients’ clinic attendance”, “Resource availability or otherwise”, and the “health worker-patient interaction”. These are depicted in Fig. 1*.*

### Patients’ clinic attendance

From the narratives, patients’ attendance and the uptake of service such as HIV testing, counselling and ART services declined during the initial phases of the pandemic. A participant observed that “Before COVID, we were seeing huge numbers each clinic day. During the pandemic, most of the clients were not reporting to the hospital.” (Nurse Maame, 2 years in ART care).

According to the participants, the most cited reason for reduced attendance to the clinic was fear of contracting the infection. A participant stated that “People [patients] were reluctant to come to the clinic … They were made to know that they were part of the high-risk group. So, some of them found it difficult to come to the clinic.” (Nurse Efua, 13 years in ART care).

From the narratives, the government strategies for reducing COVID-19 transmission such as partial lockdown in some regions and the closure of the borders also contributed to the initial decline in patients’ attendance and service uptake. Some of the participants explained that “When the President declared the partial lockdown, then everyone [patients] was like, then I have to be home, I shouldn’t go out. (Nurse Da Vinci, 3 years in ART care)

The closure of our borders because of the pandemic also affected the number of clients who used to report for treatment. This is because we have clients coming from Ivory Coast and Togo. You know that during the partial lockdown people could not travel freely from one place to the other. All these impacted on the numbers. (Nurse Maame, 2 years in ART care)

The fear and statutory declarations resulted in increased incidence of missed appointments and defaulting. The narratives revealed that “A lot of people defaulted.” (Nurse Esi, 9 years in ART care).

This decline in patients’ attendance resulted in decreased workload at the clinic during the initial days when the pandemic was announced.

Araba observed that “For the workload … I think during the COVID time it reduced; I was even asking whether the people were defaulting because of the COVID.” (Data manager Araba, 5 years in ART care).

Some participants however explained that over time, there was a surge in the number of patients after continuous efforts to track and educate defaulting clients on the need to adhere to treatment:

Sometimes we [nurses] had to call some clients to ask why they were not coming to the clinic. … Upon interacting with them, they understood that they needed the medication even amidst COVID-19. (Nurse Jessica, 2 years in ART care)

Some participants also intimated that some defaulted clients who returned after the pandemic came in deteriorated states which required increased efforts from the healthcare workers. “They [clients] defaulted and came in worse state, and we had to double our effort to bring them back to healthy state.” (Alberta, 6 years in ART care).

### Resource availability or otherwise

From the narratives, the pandemic had a dual impact on resource availability in the ART unit.

The narratives showed the pandemic affected availability of some consumables and folders which negatively impacted patient care. A participant disclosed that:

… in the initial phase of COVID-19, they started supplying nose masks and gowns … They gave us scrubs and the cloth type of nose masks (Nurse Kwesi, 2 years in ART care).

Participants also indicated adequate supply of ARVs during the early days due to push of ARVs from the national level and guidance of distribution by the Metropolitan, Municipal and District Assemblies, but recorded drug stock-out as the pandemic raged on. A nurse said “For ART drugs, we had enough until; was it, November?” (Nurse Jessica, 2 years in ART care).

The drug stock-outs resulted in reduction in the quantity of drugs supplied to the patients in most instances. Some participants explained that:

At times, the drugs will get out of stock and we [pharmacy] need to ration what is available … It came to a time, we had to do [dispense] 2 weeks and it came to 1 week.” (Dispensing Technician Akwasi, 15 years in ART)

When you [nurse prescriber] give about one month prescription to the patient and they go to the pharmacy, they may serve them only two weeks or less. (Nurse Maame, 2 years in ART care)

Strategies such as switching ARV combinations supplied to patients ensured continuity of service delivery and patients’ health were not compromised. An excerpt of a narrative read:

The clients were on different drugs [combinations] but it came to a time we were giving all of them the Dolutegravir because of shortage of those drugs. This contributed to the shortage of Dolutegravir too. (Nurse Maame, 2 years in ART care)

Some participants averred that procurement of the ARVs was affected as a result of the closure of borders across the world due to COVID-19:

In terms of procurement, we heard that the drugs are at the port but getting it is a problem and we understand that the drugs are imported from outside Ghana. There was a time that planes were not moving so all these affected the movement of the drugs to the various health facilities. (Dispensing Technician Akwasi, 15 years in ART care)

It further emerged from the data that the pandemic led to shortage of stationary. “We still don’t have folders for registration. When a patient’s folder is full, we pull out the leaves of a new one and share. So, we could share one folder between 5 patients.” (Data manager Araba, 5 years in ART care).

The narratives also revealed instances of shortage of consumables meant for COVID-19 prevention:

With the disposable gloves for example, because the organism lives on surfaces we changed often. This also led to shortage. We sometimes ran out of disinfectants. This is because we were using them more often compared to the period before the outbreak. (Nurse Maame, 2 years in ART care)

Throughout the transcripts, participants reiterated that they did not feel safe even though they were committed to providing care to patients accessing ART services. This, they mostly attributed to the non-availability of items required for COVID-19 preventive protocols.

### Health worker-patient interaction

From the narratives, it was evident that in the bid to provide care for the clients, strategies for physical and social distancing were established. Participants indicated organising the service area in conformity to the physical distancing policy outlined by the COVID-19 protocols:

We changed the sitting arrangement, we organised the consulting area so that there was a gap between us [nurses] and the patients, not like in previous times; a much wider gap … is it one or two meters? It was to ensure social distancing. (Nurse Kwesi, 2 years in ART care)

In some instances, “… the room was too small for social distancing.” (Data manager Araba, 5 years in ART care), thus creating physical distancing was sometimes a challenge:

We are free with them [patients] … so, if they have had long appointment, like four months … they even want to hug you. But now, the touching … no, no; we have stopped all those things.” (Data manager Araba, 5 years in ART care)

From the study, the measures implemented to promote safety affected the nurse-patient interaction:

Prior to COVID, we had time engaging them more. Some of them come to pour their hearts to you, so that you help them solve their problems. But because of COVID, they will tell you that they are in a hurry to go back home … So … yes, it didn’t allow us time to interact more with them as we did in the previous times. Nothing was normal anymore. (Nurse Da Vinci, 3 years in ART care)

### Theme 3: effecting change to promote safe care

From the narratives, various strategies were implemented to ensure that quality ART service delivery was maintained while promoting patient and staff safety. Three main sub-themes emerged from the analysis; “Establishing a new order”, “Promoting safety for all” and “the presumed normalcy”.

### Establishing a new order

Evidence from the data revealed that several strategies were put in place to ensure continuous service delivery during the pandemic. These strategies were built out of consensus between management and staff, while some were also based on policy directions received from the National AIDS/Control Programme (NACP)-GHS. Some of the changes to service delivery were underscored by changes in policy directions attributed to the pandemic. According to the narratives, the policy on service delivery was reviewed during the pandemic.

Concerning ART prescription and dispensing, a participant explained:

“there was a policy that those who are stable; that is; those who don't normally report ill, whose lab reports are good, and physically stable, can be given treatment for six months.” (Dispensing Technician Akwasi, 15 years in ART care)

Most of the participants cited adjusting the staff work schedules as an important strategy implemented during the period “Before COVID, we were running a single shift and seeing huge number of clients on each clinic day.” (Nurse Afia, 2 years in ART care).

The rationale was to ensure a continuous flow of staff in case a group of healthcare workers was exposed to the virus and had to be quarantined. This rescheduling also allowed periods of rest for the health staff. Nurse Afia, 2 years in ART care explained that “We [nurses] also had time to rest because we all didn’t come to work on the same clinic day.”

Adjusting the schedule was however not possible in some departments where staff numbers were few. For example, the Data Management Unit and the Pharmacy. Hence, this resulted in extended work periods for staff in those units:

Before the COVID we were closing around 2pm or 2:30pm. During COVID, we sometimes close around 3:30pm or 4pm. So COVID has extended our working time. That's one of the challenges. (Pharmacist Aaron, 3 years in ART care)

Participants also indicated that a strategy to re-structure the patient appointment schedules was implemented to ensure continuous service delivery. A participant said “During the COVID-19 period, we had limited number of people [patients] coming to clinic.” (Esi, 9 years in ART care).

The management of the appointment schedule was based on the patient’s history of honouring appointments and having achieved a suppressed viral load. A participant said:

We had to check those clients that have achieved viral suppression that is less that 1000 copies/ml. Once there is enough drug supply, we give them a duration of 6 months. This is another way of cutting down the numbers. (Nurse Da Vinci, 3 years in ART care)

Participants felt that the strategy eased up the number of persons to be seen daily and reduced the risk of exposure for both patients and healthcare workers within the unit. For Araba, a Data manager with 5 years’ experience in ART care “This was necessary … so we reduce the rate of exposure and contracting the COVID-19 [virus].”

Some participants however alluded to experiencing some challenges with the clients appointment during the pandemic when the unit experienced shortage of ARVs:

The load is increasing because they are coming back for their drugs even though their appointment is not yet due. This is because when the nurses prescribe for 6 months, they [pharmacy] give them 2 weeks so definitely they have to come back and they come and crash with those we have appointment with that day so it is making the workload increase. That is why the load is piling up again. (Data manager Araba, 5 years in ART care)

Participants also indicated that task-shifting was employed as a strategy to ease waiting time. Nurses were assigned other responsibilities to ensure that the patients were not clustered at any point along the care continuum. According to Jessica, a nurse with 2 years’ experience in ART care “Some of our nurses were also assigned to the pharmacy to assist them dispense the medication so that it wouldn’t be like all the clients have gathered there.”

Another approach employed to organise clients care was the creation of several work stations along the care continuum so that various clients was seen at the same time. This approach reportedly reduced the patients’ waiting time and facilitated fast service delivery:

We organised ourselves [nurses] into more teams for testing, medication, counselling, checking of vitals while the doctors do the consultation. So, we had about 4 nurses’ stations. When it happens that way, we realised that within averagely 10 minutes, we [nurses] can attend to four or six people [patients] at once. (Nurse Da Vinci, 3 years in ART care)

### Presumed normalcy

From the participants interviews, it emerged that as time elapse and COVID-19 was accepted as a pandemic that will persist like other diseases. Thus, efforts to ensure safety that had been established gradually waned. Some participants indicated decline in the supply of PPEs for the staff. Nurse Kwesi said “They [management] used to supply us with masks but now they don’t supply it again.”

Patient numbers also increased markedly “Before the second wave, when the situation was normalising, the patient numbers increased again. (Nurse Esi, 9 years in ART care).

Also, there was evidence of a setback in some patients’ commitment to protect themselves, other patients and healthcare workers:

Most of the clients are not cooperative … putting on the mask throughout maybe 2 or 3 hours that they will spend in the hospital is a bit challenging. Sometimes, you even have to go to the extent of saying that, if you don’t wear the mask then I am not attending to you. (Nurse Jessica, 2 years in ART care).

From the data however, some health care workers routinised the COVID-19 protocols and continued to observe them even though they accepted that COVID-19 had come to stay:

For now, we are trying to do our best; after every hour or 2 hours, I have to go and wash my hands, sanitize it, come back and handle the folders. I am doing the protocols just to psyche my mind that I am okay. (Pharmacist Aaron, 3 years in ART care)

## Discussion

The study found that at the onset of COVID-19 pandemic, healthcare workers experienced fear because of the anticipated risk of infection to themselves and their immediate families while working with patients who were noticeably at high risk. This reaction may not be unfounded as Ashinyo and colleagues reported a high level of exposure and risk (80.4%) of COVID-19 among healthcare workers in a Ghanaian hospital [15]. Similar evidence of psychological effects of COVID-19 on healthcare workers such as psychological distress, depression and anxiety uncovered in both developing and developed countries such as Ethiopia and Pakistan [16, 17], and China and United Kingdom [8, 18–20]. Although, the study found that the health system made efforts to reduce the risk of exposure, there was no indication of efforts directed as managing the impact the pandemic had on the psychological wellbeing of the health staff. Targeted efforts such as reflective counselling and resilience-focused education [18, 21] can enhance coping management among the healthcare workers providing HIV care.

In this study, decrease service uptake including HIV testing, missed appointments and defaulting were recorded as a result of the COVID-19 pandemic. Although, numerous studies reported similar occurrences among PLHIV before the pandemic [22, 23], factors underscoring the low service uptake were different; fear of infection and statutory declarations of lockdown. These lockdowns and imposition of restrictions were not limited to the Ghanaian society but extended across several countries [12] and reportedly impacted access to healthcare services [24]. In this study, the restrictions resulted in shortage of test kits and ARVs as a result of delayed in-country shipments and port clearing. The lower rate of HIV testing as well as missed refill appointments could infer late initiation of treatment and incomplete adherence respectively which has been attributed to increased viral load and risk of transmission [25]. The implications of COVID-19 pandemic on Ghana’s ability of achieving the global target of 90–90-90 can therefore not be overlooked [26].

The study also found that COVID-19 protocols including frequent handwashing and sanitization, wearing of mask and social distancing was implemented in the ART unit. This finding is in congruence with another study by [27] that reported a significantly high adherence to COVID-19 protocols in a German hospital. It is likely that the healthcare workers increasing access to knowledge about the mode of transmission impacted their compliance with the protocols. But other safety strategies such as physical distancing and reduction in waiting time also impacted the healthcare worker-patient relationship negatively. Several studies have alluded to the fact that interactions between healthcare workers especially nurses and PLHIV have positive implications on the psychological wellbeing of the clients and their adherence to treatment [28]. There is therefore the need to adopt policies to enhance the client-healthcare worker interactions while social and physical distancing is being encouraged.

In this study, several strategies were applied to ensure continuity of service delivery while promoting patient and staff safety. Task-shifting ensured continued service delivery during the pandemic. Task-shifting has been recommended by WHO as a strategy to curb the effects of shortage of skilled personnel to provide the ART care and to enhance accelerated rolling out of ART services [29]. As in most resource poor settings where task-shifting is practiced in ART care [30, 31], the strategy was effective in this setting during the pandemic as it allowed for staff schedules to be restructured and reduced the waiting time.

Additionally, the study found the institution of an ART prescription and dispensing schedule that enabled multi-month prescription refills for stable clients. This strategy is in line with a recommended Differentiated Care Model that proposed one clinical visit every 6 months with quarterly ARV refills for stable clients [26]. During this pandemic, the model presents an opportunity to reduce the burden on congested health systems and decrease the costs incurred by clients as a result of the number of clinic visits for ART refill, thereby enhancing both staff and patients’ safety [32, 33]. Although initially successful, the strategy had to be shelved as the facility experienced ARV shortages during the pandemic. This disruption in ART supply and distribution during the pandemic was projected by several modelling studies [34, 35]. Further to this, UNAIDS reports that the pandemic has caused disruptions in health service delivery and unequal access to ART globally, and therefore anticipates that HIV targets set for 2020 may not be achieved by most countries [12, 36]. The study found that to ensure continuous ART service delivery in the wake of the ARV shortage, healthcare workers had to revert to the status quo where clients had to make several visits to the clinic for ART refill by limiting the quantities dispensed or changing the treatment combination all together although this was against the NACP guidelines.

A strength of the study includes its originality. The design allowed for an in-depth exploration of the topic under study. It also allowed for a thick description of the perspectives of the healthcare workers providing HIV services on how COVID-19 is impacting service delivery.

Furthermore, recall bias could have occurred as the participants were expected to share experiences that may have occurred more than 6 months. The researchers however, tried to minimize this by using clarifications and feedback interviews as well as member checking to allow for the participants to report their experiences as close to the actual as possible.

It is recommended that further study is conducted in the area the impact of COVID-19 on HIV care from the patients’ perspective and strategies to enhance the coping strategies of healthcare workers providing ART care during the pandemic.

## Conclusion

The COVID-19 pandemic impacted HIV service delivery in this study setting. It affected the healthcare workers psychologically and impacted service delivery. Although several strategies were implemented to manage the effects of the pandemic on ART care, there is a need to establish of pathways of support such as reflective counselling for healthcare workers within the ART clinic and to consolidate as well as institutionalise the change that ensure continuous service delivery.

## Data Availability

All data generated or analysed during this study are available from the corresponding author on reasonable request.

## Abbreviations

ART: Antiretroviral Therapy
ARV: Antiretrovirals
HIV: Human Immunodeficiency Virus
NACP: National HIV/AIDS Control Programme
PLHIV: Persons Living with HIV
UNAIDS: Joint United Nations Programme on HIV/AIDS
WHO: World Health Organization
